# The Association of Outpatient Cost-Sharing Policy with Health and Economic Outcomes for Rural Children in China: A Cross-Sectional Study

**DOI:** 10.3390/healthcare14010063

**Published:** 2025-12-26

**Authors:** Chen Wu, Lixiong Yang

**Affiliations:** School of Labor and Human Resources, Renmin University of China, Beijing 100872, China; chenw@ruc.edu.cn

**Keywords:** outpatient cost-sharing, rural children’s health, medical expenditure, health equity

## Abstract

**Highlights:**

**What are the main findings?**

- The outpatient cost-sharing policy is associated with better health outcomes for rural children. This relationship appears to be linked to three potential factors: higher utilization of outpatient services, better health of mothers, and greater school-related food and accommodation expenses.
- The core value of the policy lies in including high-frequency outpatient expenses in the reimbursement scope. Unlike findings for adults, it is not associated with reduced hospitalization rates, suggesting that pediatric inpatient demand is rigid and non-discretionary.

**What are the implications of the main findings?**

- The policy’s design is compatible with the specific healthcare needs of children, which may inform policymakers to expand the policy to include more rural children.
- Policy evaluation may need to be differentiated for children and not assume adult patterns (like inpatient substitution).

**Abstract:**

**Background/Objectives**: Under the urban–rural dual structure, rural children’s health security faces multiple challenges. These stem from geographical disadvantages, inadequate resources, and systemic flaws in medical insurance design. The outpatient cost-sharing policy is a key design to address these issues. **Methods**: Using data from the 2018 China Household Income Project (CHIP), this study employs Propensity Score Matching, Ordered Probit, Logit, and a Two-Part Model to examine the association between the policy and the health and economic outcomes of rural children. **Conclusions**: The results show that the policy is significantly associated with better child health scores and a higher probability of reimbursement. These positive associations appear to be connected to three potential factors: higher use of outpatient services, better mother’s health, and greater school-related food and accommodation expenses. In contrast to adult populations, no significant substitution between outpatient and inpatient services was observed for children, suggesting the non-discretionary and rigid nature of pediatric hospitalization decisions. This research provides robust empirical evidence for the policy’s potential benefits, offering important implications for optimizing the child medical security system.

## 1. Introduction

Investment in childhood health is a cornerstone of sustainable population development, generating significant social returns and lifelong benefits [1]. However, China’s urban–rural dual structure jeopardizes rural children’s health through geographical disadvantages, resource misallocation, and institutional flaws, rendering them a vulnerable group in the “Healthy China” initiative. First, a significant structural imbalance exists in the supply of quality pediatric resources between urban and rural areas. Rural regions suffer from a shortage of qualified pediatricians, insufficient primary care capacity, and a lack of advanced diagnostic equipment. Consequently, rural families face significant barriers—including travel distance, time, and transportation costs—to accessing care comparable to urban standards. This supply-side deficiency creates economic burdens and dilemmas, forcing households to choose between inadequate local care and costly treatment in distant hospitals.

Concurrently, the protection provided by basic medical insurance for children is markedly insufficient. The main public medical insurance for children in China is the Urban and Rural Resident Basic Medical Insurance (URRBMI) system, which applies the same financing and benefit standards to them as to non-employed adults. The system exhibits a design bias toward covering major illnesses and inpatient services. However, research indicates that children are predominantly affected by common conditions such as respiratory and digestive illnesses, characterized by an “outpatient-heavy and inpatient-light” utilization pattern [2]. This mismatch between insurance design and children’s healthcare needs results in a low effective reimbursement rate for outpatient costs. One study estimated that the average out-of-pocket share of medical expenses for rural children reached 92.1% [3]. This combination of inadequate supply and misaligned insurance increases child-rearing costs and threatens the long-term health of rural children.

In this context, the basic medical insurance’s outpatient cost-sharing policy, which aims to strengthen primary care capacity and reduce the public’s outpatient financial burden, offers a precise policy instrument to address the aforementioned predicaments faced by rural children. This policy incorporates high-frequency, low-cost outpatient expenses into the social pooling fund. It shifts these costs, previously paid out-of-pocket, to a risk-pooling model. Furthermore, it enhances reimbursement levels at primary healthcare institutions to optimize medical resource allocation. This policy design directly and precisely addresses the dual squeeze: on one hand, it lowers the out-of-pocket costs for routine outpatient visits, creating economic incentives for rural families to seek timely medical care, ensuring that children receive continuous and timely basic medical services. On the other hand, by setting higher reimbursement rates at primary care facilities, the policy effectively encourages utilizing grassroots care as the first point of contact. This not only provides a more convenient and affordable healthcare option for rural families burdened by travel and other non-medical costs but, in the long run, also helps to channel insurance funds and patient flows to the grassroots level, thereby feeding back to and bolstering the service capacity and quality of rural medical institutions. Since the establishment of the catastrophic-illness pooling plus outpatient cost-sharing model in 2008, the policy has continuously evolved. Local implementation rules have explicitly designated children as a key priority group, generally granting them favorable benefits (e.g., lower deductibles, higher reimbursement rates), reflecting a policy orientation aimed at bridging the last mile in rural children’s health security.

The implementation of China’s outpatient cost-sharing policy exhibits regional heterogeneity, which correlates with factors such as local economic development levels. Policy documents from major regions show that these disparities appear primarily in three dimensions: deductibles, reimbursement ratios, and payment ceilings. First, concerning deductibles, regions differ regarding the presence or absence of a threshold. Provinces such as Jiangxi and Hunan, along with certain municipalities, have not established deductible lines. Conversely, regions like Beijing and Shanxi have set differentiated deductibles based on the tier of the medical institution. While this design supports the hierarchical medical system, it may discourage the use of some low-cost outpatient services. Second, regarding reimbursement ratios, regions generally adopt differentiated payment strategies that favor primary care institutions. Although some regions implement a uniform reimbursement ratio, the majority (e.g., Tianjin, Shanxi, and Henan) stipulate significantly higher ratios for primary medical institutions compared to secondary and tertiary hospitals, with differentials typically ranging from 5% to 25%. This price leverage mechanism aims to incentivize rural households to prioritize village clinics or township health centers for common childhood diseases, thereby promoting tiered diagnosis and treatment. Finally, significant disparities exist across regions regarding maximum payment limits. In economically developed regions (e.g., Beijing, Tianjin, and certain cities in Jiangsu), annual ceilings can reach several thousand yuan, effectively covering the annual outpatient medical risks for children. In contrast, in parts of the central and western regions (e.g., certain cities in Gansu and Sichuan), annual payment limits may be as low as 80 to 200 yuan, or are subject to per-visit (or daily) caps.

A rich body of academic literature has explored the effects of the basic medical insurance outpatient cost-sharing policy. The general consensus is that the policy effectively enhances access to and utilization of outpatient services for the insured, particularly for vulnerable groups such as low-income individuals and those with chronic diseases [4,5,6,7]. However, a central and unresolved debate in current research is whether increased service utilization necessarily translates into substantive improvements in health outcomes and effective control of medical costs. On one hand, some studies have confirmed the policy’s positive effects. Timely outpatient care can effectively manage medical conditions and prevent minor ailments from escalating into major diseases, thereby directly improving the health of farmers and middle-aged and elderly employees [8,9]. It may also generate overall cost savings by substituting for more expensive inpatient services [10]. On the other hand, some research has raised doubts or uncovered complexities regarding the policy’s effects. For example, the policy may induce ex-post moral hazard, wherein patients are inclined to consume more low-value care services that offer limited health benefits, thereby straining the insurance fund [11]. The mixed (positive or negative) empirical findings in the existing literature are deeply rooted in the heterogeneity of policy design (e.g., benefit levels, whether services are restricted to primary care facilities) and variations in study populations, causing different policy parameters to have disparate impacts on the health and economic burdens of different groups [12]. For instance, one study pointed out that overly limited benefit levels can create a conflict between insured people’s psychological expectation of being covered and the reality of inadequate protection, potentially even exerting a negative impact on their perceived mental health [13].

Existing literature, whether supportive or critical, has focused almost exclusively on adults, particularly the elderly, employees, and patients with chronic diseases. However, existing findings regarding policy effects on adults—such as the “substitution effect” or “moral hazard”—are difficult to directly extrapolate to the pediatric population. This is mainly due to fundamental differences between the two groups. Key differences include health production functions, price elasticity of demand, and medical decision-making [14,15]. For instance, in contrast to the prevalence of chronic conditions among adults, pediatric diseases are predominantly acute respiratory or digestive tract infections. These conditions are characterized by sudden onset and rapid progression, the corresponding medical services exhibit “high marginal health benefits” and “low price elasticity of demand.” Therefore, the likelihood of induced demand (moral hazard) arising among children is relatively low; instead, the risk of health loss resulting from underutilization warrants greater attention [16,17]. Consequently, investigating the association between outpatient cost-sharing policy and the health and economic outcomes of rural children not only fills a significant gap in the existing literature but also provides precise empirical evidence tailored to pediatric characteristics. Ultimately, this serves to rectify potential biases inherent in directly transplanting adult-centric policy logic onto the child population.

The contributions of this study are threefold: First, by shifting the research focus from the general population to rural children, it fills a critical gap in the literature regarding the policy’s benefits and potential pathways for a key vulnerable group. Second, it provides more comprehensive evaluative evidence by simultaneously examining the policy’s relationships with both health outcomes and economic burden. Finally, the findings will offer important policy implications for optimizing China’s child medical security system. In conclusion, this study not only theoretically elucidates the relationship between outpatient cost-sharing policy and rural children, but also provides key micro-evidence for the practice of utilizing medical insurance design to interrupt the inter-generational transmission of “health poverty” and support China’s rural revitalization.

### 1.1. Theoretical Framework

Unlike other age cohorts, children are unable to make independent decisions regarding their own health investments due to their developmental immaturity. Consequently, child health is incorporated into the family utility function, with parents or other family members making relevant medical decisions on their behalf [18]. To this end, this paper first constructs a family utility function. This function treats the family as the decision-making unit, aiming to maximize family utility:

(1) $$U=U(H_{child},H_{mother},Z)$$

While many factors influence the family utility function, this study focuses on the child’s health status $H_{child}$ and the mother’s health status $H_{mother}$, with other household consumption goods represented by the variable Z.

Families do not purchase child health directly; instead, they “produce” it by inputting various factors. Its production function can be expressed as:

(2) $$H_{child}=f(M_{child\_outpatient},M_{child\_inpatient},N_{child};H_{mother},E)$$

where $M_{child\_outpatient}$ represents the utilization of outpatient medical services for the child; $M_{child\_inpatient}$ represents the utilization of inpatient medical services; and $N_{child}$ represents other health-related inputs for the child, such as food supplements. $H_{mother}$ and E denote the mother’s health status and other household endowments, respectively. The first three variables are regarded as direct production factors of the child’s health status, while the latter two variables are viewed as key inputs influencing the efficiency of child health investment. The healthier the mother, the higher the efficiency with which direct production factors are converted into child health [19].

Rational households aim to maximize utility within a budget constraint. This paper introduces the constraint function as follows:

(3) $$P_{z}Z+P_{n}N_{child}+P_{o}(1-r_{o})M_{child\_outpatient}+P_{i}(1-r_{i})M_{child\_inpatient}+w_{t}+S(\sigma^{2})\leq W$$

Here, the total household income W is allocated across various expenditures. The variables $P_{z}$, $P_{n}$, $P_{o}$, $P_{i}$ represent the prices of other consumption goods, non-medical health-related products, outpatient services, and inpatient services, respectively. The variable $w_{t}$ denotes the opportunity cost for rural households to obtain medical services for children, including transportation and caregiving costs. The variables $r_{o}$ and $r_{i}$ represent the reimbursement ratios for outpatient and inpatient services, respectively. The variable $S(\sigma^{2})$ represents the household’s current precautionary savings, the magnitude of which is directly proportional to the risk $\sigma^{2}$ faced by the household.

### 1.2. Research Hypotheses

This paper argues that the key to understanding the effects of the outpatient cost-sharing policy lies in recognizing the fundamental differences in healthcare demand between rural China and developed nations. In the healthcare systems of developed countries, represented by the United States, increasing copayments is typically viewed as a mechanism to curb excessive medical care and moral hazard; its policy objective is to suppress utilization rates without compromising health outcomes [20,21]. However, within the context of China’s urban–rural dual structure, the primary challenge facing rural children is not “over-utilization,” but rather “under-utilization” constrained by affordability and the unequal distribution of medical resources [22,23,24]. Consequently, within the context of this study, reducing copayments (i.e., increasing reimbursement ratios) should not be construed as an inducement for moral hazard, but rather as a form of “pro-social investment in human capital.” In other words, one of the objectives of implementing the current outpatient cost-sharing policy in China is to achieve better health outcomes for rural children, rather than cost containment or the prevention of excessive medical care.

This paper posits that the outpatient cost-sharing policy will be positively associated with children’s health. First, the policy lowers the price of outpatient care by increasing the reimbursement rate or expanding reimbursement coverage, and reduces opportunity costs (like travel for rural children) by incentivizing local care. This may encourage higher utilization of outpatient services, leading to more regular and timely treatment which is conducive to improving child health. Secondly, by lowering medical costs, the policy might also relate to the mother’s health status [25]. Since mother’s health is a key input for the efficiency of child health production, this could indirectly boost the child’s well-being. Thirdly, if the policy reduces out-of-pocket spending or lowers psychological expectations of future medical expenses, it frees up household income. This allows families to reallocate resources toward other health inputs, such as school-related food and accommodation expenses [26].

The policy’s relationship with total out-of-pocket medical expenditure of children is uncertain. This ambiguity arises from two effects. First, the association with outpatient spending is unclear: while the quantity of services will rise, total expenditure depends on the price elasticity of demand [27], which is unknown for this suppressed-demand population. Second, the policy may create a substitution effect: better access to outpatient care (early treatment) can reduce the need for expensive inpatient services [28]. The final change in total costs depends on the trade-off between the change in outpatient spending and the cost savings from inpatient substitution.

Based on the theoretical derivations above, this paper proposes the following hypotheses for empirical testing:

**Hypothesis** **1.** *The outpatient cost-sharing policy is positively associated with the health status of rural children.*

**Hypothesis** **1a.** *The positive association between the policy and rural children’s health may be partially associated with better health status of their mothers.*

**Hypothesis** **1b.** *The positive association between the policy and rural children’s health may be partially linked to greater school-related food and accommodation expenses.*

**Hypothesis** **2.** *The policy is associated with a significantly greater probability of outpatient service utilization and a decreased probability of inpatient service utilization for rural children. Its association with children’s out-of-pocket medical expenditure is uncertain.*

## 2. Data and Methods

### 2.1. Data Source and Variable

This paper uses data from the 2018 China Household Income Project (CHIP), conducted by Beijing Normal University. CHIP is a large-scale nationwide household survey project conducted separately for urban and rural areas. The survey sample is drawn from the large-scale sample pool of the routine household survey of the National Bureau of Statistics (NBS) of China, based on two principles: first, regional representativeness, whereby China’s 31 provinces are divided into three regions (Eastern, Central, and Western), with representative provinces selected from each; and second, random sampling, whereby sub-samples are selected from the large sample strictly adhering to random principles to ensure adequate coverage of the involved provinces. Since we focus on the outpatient cost-sharing system for rural children, we selected rural household survey data from CHIP. The sampling strategy followed a stepwise exclusion procedure to ensure data quality. Initially, the dataset included all rural individual samples. We restricted the sample to children aged 0–14, which aligns with the standard definition of children in health economics. Subsequently, we excluded individuals who did not participate in the New Rural Cooperative Medical Scheme (NRCMS) or the Urban and Rural Resident Basic Medical Insurance (URRBMI) to ensure the study focused on the insured population relevant to the policy. Ultimately, a valid sample of 3123 individuals was obtained, covering 124 cities across 13 provinces.

The dependent variables in this paper are of two types. The first category measures the health status of children, including a health score (5 = very good, 4 = good, 3 = fair, 2 = poor, 1 = very poor), which is derived from the questionnaire item: “Compared to peers, what is your current health status?” (answered by parents or guardians). The second category measures children’s medical service consumption, including the children’s out-of-pocket medical expenses in the past year, which were subjected to logarithmic transformation calculated as ln(expenses + 1); and children’s medical service reimbursement ratio, calculated as the reimbursement amount divided by total medical expenses multiplied by 100.

The core independent variable of this study is whether the outpatient cost-sharing policy is implemented. This paper compiled the policy implementation status for the 124 cities covered in the sample by searching government websites. A binary variable was constructed: if the sample location had effectively implemented the outpatient cost-sharing policy on or before 1 January 2018, it was assigned a value of 1; otherwise, it was assigned a value of 0. As of 1 January 2018, 56 cities had implemented the policy, comprising 1418 observations; 68 cities had not, comprising 1705 observations. Specific details are provided in Appendix A, Table A1.

Existing literature indicates that child health is influenced by prenatal development, family, social, and economic environments [29]. This paper selects a rich set of control variables at the individual, household, and city levels. Individual-level controls include age, gender, and birth weight (unit: jin [0.5 kg]). Household-level controls include the mother’s employment status (0 represents unemployed, 1 represents employed) and household per capita income (in 10,000 RMB). The city-level control variable is the logarithm of the number of hospital beds per 10,000 people. Furthermore, given the significant regional disparities across Chinese provinces, province-level fixed effects were included to control for potential confounding factors at the regional level [30].

It is important to note that many policies in China often follow the logic of “rich first, poor later” and “pilot first, promotion later” [31]. The outpatient cost-sharing policy is no exception; its adoption and implementation across cities is not a random process. Generally, regions with better economic foundations and more comprehensive healthcare systems are more likely to be the first to launch outpatient cost-sharing policies to improve the sense of gain among insured residents. This “non-random” policy intervention implies that cities implementing the policy may be superior to non-implementing cities in terms of social-economic characteristics and medical resource endowments. To visually demonstrate this disparity, we compared the differences between the treatment group and the control group across relevant dimensions and conducted t-tests; the results showed that the treatment group possessed higher household per capita income, more beds per 10,000 people, and a higher rate of mother employment (see Appendix A, Table A2).

This paper examines the potential factors associated with the relationship between outpatient cost-sharing policy and children’s health and medical expenditure by analyzing mother’s health status, children’s school-related food and accommodation expenses, and the probability of outpatient and inpatient visits. Mother’s health status is measured using a health score, consistent with the child health score variable, and the sample is restricted to mothers who participated in the New Rural Cooperative Medical Scheme or Residents’ Insurance and lived with the child. Children school-related food and accommodation expenses is measured by the logarithm of food and accommodation expenses within the family’s education expenditure for the child in the past year (including on-campus food and accommodation, and possible off-campus living expenses for schooling). The variables for outpatient and inpatient probability are binary indicators for whether the child had an outpatient visit or was hospitalized in the last three months. Table 1 presents the descriptive statistics of the variables used in this paper.

### 2.2. Methods

As previously noted, while the macro-institutional frameworks and policy objectives differ between China and Western developed nations, the micro-level decision-making logic of individual households—specifically, the maximization of child health utility subject to budget constraints—possesses cross-cultural universality. Classical economic models serve essentially as mathematical descriptions of this universal human behavior (i.e., responsiveness to price incentives) rather than as artifacts of any specific political system. This paper applies these standard economic models to investigate and demonstrate the relationship between China’s outpatient cost-sharing policy and the health and economic outcomes of rural children in China.

#### 2.2.1. Baseline Model and Endogeneity Issues

To mitigate the aforementioned “selection bias” arising from the non-random implementation of the policy, this study employs the Propensity Score Matching (PSM) method. The core rationale of PSM is to identify one or more counterfactual counterparts in the non-participating group for each child in the treatment group who are highly similar across all observable characteristics, thereby mitigating bias through these counterfactual comparisons. This study incorporates key determinants of policy adoption, including city-level medical resource supply (number of beds per capita) and household-level socioeconomic status (household per capita income, mother employment status, etc.). These variables encompass the primary dimensions influencing local government policy decision-making and household demand for medical care. We maintain that after controlling for these key covariates and passing the PSM balance test, systematic differences between the treatment and control groups can be substantially and effectively balanced, thereby mitigating “selection bias”.

It is important to acknowledge that PSM can only adjust for biases stemming from observed characteristics included in the model, and cannot remove bias arising from unobserved confounding factors, such as unmeasured heterogeneity at the city or household levels.

The process involves three steps. First, estimate the propensity scores. This paper uses Logit model, with policy participation as the dependent variable and all control variables as independent variables, to estimate the conditional probability of participation for each sample. Second, based on the propensity scores, the matching is performed using the Kernel matching with a default bandwidth of 0.06, and is restricted to the region of common support. And a caliper constraint, which is one-quarter of the standard deviation of the estimated propensity scores, is added. To ensure the quality of the match, the paper will rigorously test whether there are significant differences in the control variables between the two groups after matching, require that the standardized bias (% bias) of all variables after matching is less than 10%, and all t-test results accept the null hypothesis that there is no systematic difference between the treatment group and the control group, ensuring the balancing assumption is satisfied. Finally, a weighted regression analysis is performed on the matched sample to obtain robust estimates of the policy association with health outcomes.

#### 2.2.2. Health Model

This paper employs an Ordered Probit model to examine the relationship between the policy and rural child health. In this study, the core dependent variable, “child health score,” is an ordered categorical variable ranging from 1 to 5. Although these figures appear numerical, they represent rank order rather than cardinal values. For such variables, traditional linear regression models are inappropriate. The Ordered Probit model is a regression technique specifically designed to handle ordered categorical variables. Therefore, we used the Ordered Probit model for estimation.

This model assumes an unobservable continuous latent variable $Y_{i}^{*}$ representing the child’s true health status, with the determining equation:

(4) $$Y_{i}^{*}=\beta_{1}{\mathrm{Policy}}_{i}+\beta_{2}X_{i}+\mu_{i}$$

The relationship between $Y_{i}^{*}$ and the observed health score $Y_{i}$ is determined by a set of thresholds $\tau_{k}$:

$$Y_{i}=k\;{}^{\circ}\;if\;{}^{\circ}\;\tau_{k-1}<Y_{i}^{*}\leq \tau_{k}$$

where k represents the health level. Combined with the PSM method, this paper will conduct a weighted Ordered Probit regression on the matched sample to estimate the association of outpatient cost-sharing with the probability of improving children’s health.

#### 2.2.3. Medical Expenditure Model

This paper uses Two-Part Model (TPM) to evaluate the relationship between policies and rural children’s medical expenses. Children’s medical expenditure data has two typical characteristics: a large number of zero values (many children incurred no medical expenses during the observation period) and a significant right-skewed distribution of the positive values. TPM decomposes the medical expenditure decision into two independent processes:

(5) $$E\left(\ln m_{i}|X_{i}\right)=P\left(m_{i}>0|X_{i}\right)E\left(\ln m_{i}|m_{i}>0,X_{i}\right)$$

The first part is the healthcare-seeking decision model, which mainly answers whether medical expenses have occurred. This paper uses the Logit model to estimate whether children experience positive medical expenses during the observation period. The second part is the expenditure level model, which mainly answers the question of “how much is spent if expenses are incurred”. This paper uses the linear regression model to estimate positive medical expenses.

#### 2.2.4. Medical Service Utilization Model

To further investigate the policy’s relationship with specific medical behaviors, this paper uses whether a child had an outpatient visit or was hospitalized in the last three months as the dependent variables. These are typical 0–1 binary variables, for which Logit model is used:

(6) $$P\left(Y_{i}=1|X_{i}\right)=\frac{\exp\left(\gamma_{1}{\mathrm{Policy}}_{i}+\gamma_{2}X_{i}\right)}{1+\exp\left(\gamma_{1}{\mathrm{Policy}}_{i}+\gamma_{2}X_{i}\right)}$$

All models in this paper employ standard errors clustered at the city level. Additionally, all models incorporate the full set of control variables at the individual, household, and city levels, and include province-level fixed effects [30]. Sampling weights were not applied in models, as we prioritized the internal comparability between the treatment and control groups over descriptive population representativeness. Furthermore, sampling weights may reduce estimation efficiency. Statistical analyses were performed using Stata/SE version 16.0 (StataCorp LLC, College Station, TX, USA). These specifications will not be reiterated hereafter.

## 3. Results

This section tests the research hypotheses using the specified empirical models, reporting baseline results on rural children’s health and medical expenditure, followed by an analysis of potential factors associated with these outcomes.

### 3.1. Baseline Results

Table 2 reports the baseline results. In the full-sample Ordered Probit model (Column 1), the policy coefficient is positive but statistically insignificant.

To address endogeneity, we employ Propensity Score Matching (PSM). First-stage estimates (Appendix A, Table A3) show that household income, medical resource availability, mother’s employment, and child characteristics significantly relate to policy implementation, indicating that policy implementation is not a random process, underscoring the importance of eliminating such selection bias via propensity score matching.

Column (2) presents the estimation results based on the PSM-matched sample, where the policy coefficient becomes significant (0.307, *p* < 0.05). The marginal effect indicates that the policy is associated with an 11.2 percentage-point increase in the probability of children reporting “very good” health. This finding is consistent with Hypothesis 1.

**Figure 1:**
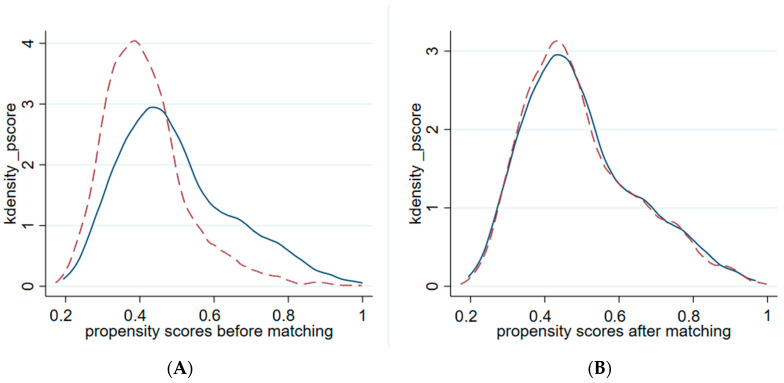
Propensity Score Distribution. Note: Solid lines represent the treatment group; dashed lines represent the control group.

In Figure 1, panel A depicts the kernel density estimation of the propensity scores for the treatment group (solid line) and the control group (dashed line) before matching. Panel B displays the distribution after performing Kernel matching. The substantial overlap in Panel B indicates that the matching procedure successfully balanced the covariate distribution between the two groups, satisfying the common support assumption. We also examined the balance of all covariates before and after matching based on standardized percentage bias (%bias) and *t*-tests. Prior to matching, significant imbalances existed across several key characteristics. Specifically, variables such as age, mother’s employment, HH per capita income, and hospital beds exhibited statistically significant differences (*p* < 0.05). Notably, the standardized bias for hospital beds and HH per capita income was substantial (42.3% and 37.8%, respectively), suggesting strong selection bias in the raw sample. After applying Kernel matching, the standardized bias for all covariates was reduced to below 5% (with a maximum of 3.8%). Furthermore, post-match t-tests for all variables yield statistically insignificant results (*p* > 0.1), indicating that the treatment and control groups are indistinguishable in terms of observable characteristics. These results confirm that the balance condition is well satisfied.

Table 3 employs a Two-Part Model to analyze medical service expenditure and reimbursement ratios. The “first part” captures the probability of incurring expenses or receiving reimbursement, while the “second part” measures the specific amounts or ratios for those who did.

First, regarding out-of-pocket (OOP) expenditure, both the first (−0.050) and second stage (−0.257) coefficients are insignificant (Table 3, Columns 1–2). This indicates that the policy is not significantly associated with the probability or amount of OOP expenditure for rural children.

Second, for the reimbursement ratio (Columns 3–4), the first-stage coefficient is significantly positive (0.594, *p* < 0.05), with an average marginal effect of 8.9 percentage points, which means that the policy is associated with an increase in the probability of rural children receiving reimbursement by an average of 8.9 percentage points. The second stage is insignificant. This indicates that, for those rural children already enjoying reimbursement, the policy is not significantly associated with the specific value of their reimbursement ratios. These results are consistent with Hypothesis 2.

In economic terms, these results may reflect an association at the extensive margin: the policy appears to be linked to a lower threshold for accessing outpatient security, incorporating a larger group of children into the reimbursement scope. However, the intensive margin appears to be limited: the policy is not significantly associated with children’s overall OOP expenditure, nor does it show a major link with overall reimbursement levels. This suggests the policy’s primary function appears to be expanding coverage breadth—enabling reimbursement for minor outpatient issues (e.g., common colds)—rather than reducing costs.

### 3.2. Robustness Checks

#### 3.2.1. Sample Sensitivity Analysis

To check results are not driven by a single city with an overwhelmingly large sample size, we excluded Tianshui (the largest sample city, 163 observations) and re-estimated the models. The associations with health, expenditure, and reimbursement remained stable (Appendix A, Table A4, Columns 1–3), consistent with baseline findings.

#### 3.2.2. Alternative Econometric Models

We employed PSM-OLS and Tobit models (suitable for zero-inflated expenditure data) as alternative models (Appendix A, Table A4, Columns 4–6). The results align with the baseline.

#### 3.2.3. Rosenbaum Bounds

Propensity Score Matching (PSM) accounts only for selection based on observables; thus, potential unobserved confounders at the city or household level may still bias the estimates. So we conducted a Rosenbaum bounds sensitivity analysis to assess the robustness of the PSM to unobserved factors and determine whether the potential hidden bias is within an acceptable range. The results show that the maximum Gamma value is 2.1 at the 0.10 significance level. This indicates the reliability of our findings.

### 3.3. Exploratory Analysis

This section examines potential factors connected to the relationship between the policy and the health outcomes of rural children: mother’s health, school-related food and accommodation expenses, and medical-seeking behavior.

Notably, it is crucial to acknowledge the limitations imposed by the cross-sectional nature of our data. Specifically, we lack the temporal ordering required to formally establish mediation or causal chains (e.g., verifying that improvements in maternal health occurred chronologically after policy implementation but before child health improvements). Therefore, the following analysis should be interpreted as descriptive associations that are consistent with theoretical expectations, rather than as definitive causal mechanisms or proven pathways.

Columns 1–2 of Table 4 show that the policy is significantly associated with better mother’s health. The marginal effect result shows that the policy is associated with a 7.9 percentage-point increase in the probability of mother’s health status being “very good”. This is consistent with Hypothesis 1a. As primary caregivers, healthier mothers may be associated with higher child health level by health production function and positive intergenerational spillovers.

Regarding school-related food and accommodation expenses, the policy is not associated with the probability of incurring these expenses (Column 7, Table 4), but is significantly associated with higher expenditure for those with positive spending, reflecting a 26.1% increase (Column 8, Table 4). This finding is consistent with research Hypothesis 1b. As a critical health-related expenditure in children’s daily school life, an increase in school-related food and accommodation expenses may be associated with better child health outcomes. Specifically, given its nature as a relatively rigid expenditure, such an increase may be interpreted as a form of parental “targeted investment” and preference, and could also suggest greater spending on unmeasured health-related items (such as hygiene products and clothing).

Columns 3–6 in Table 4 analyze medical-seeking behavior. The result of Column 4 shows that the coefficient is significantly positive, with an average marginal effect of 6 percentage points, which means that the policy is associated with an increase in the probability of rural children seeking outpatient care by an average of 6 percentage points. This finding aligns with the expectation of Research Hypothesis H2.

In the column 5–6 of Table 4, the coefficient of the policy variable is negative but statistically insignificant, indicating that the policy has no significant association with the probability of hospitalization. This contrasts with the statement about “the policy is associated with a significantly decreased probability of inpatient service utilization” in hypothesis 2. This may indicate that the expected substitution effect between outpatient and inpatient services in hypothesis H2 may be very weak in practice (this substitution effect has been observed in many studies on adult or elderly).

One plausible explanation for this phenomenon lies in the rigidity of pediatric hospitalization decisions. Specifically, this rigidity manifests at both the clinical characteristics and decision-making model levels. Clinically, pediatric hospitalizations often involve acute, rapid-onset conditions (e.g., severe infections) where outpatient care serves as screening rather than prevention, unlike the chronic disease management effective for the elderly [32]. Decision-wise, pediatric care relies on risk-averse parents who lack the ability and Information to negotiate medical recommendations, contrasting with adults who can weigh trade-offs based on experience [33]. This may explain why a policy that effectively guides the medical behavior of the middle-aged and elderly might not be associated with similar patterns in the pediatric population.

**Figure 2:**
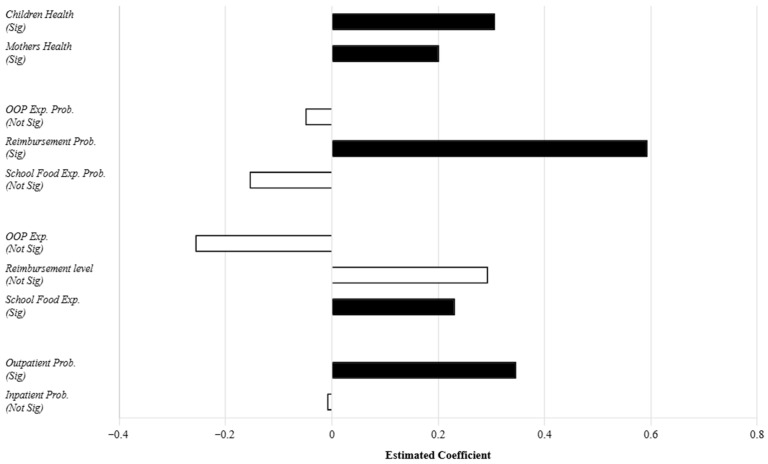
Summary of Policy Associations across Outcomes. Note: Solid black bars denote statistical significance (*p* < 0.1); Hollow bars denote insignificance.

Figure 2 summarizes the estimated coefficients of the outpatient cost-sharing policy across key outcome variables. Notably, the contrast between greater outpatient visits and stable out-of-pocket expenditure probability may suggest the policy relates to the “site of care” rather than the “decision to seek treatment.” One plausible explanation is that, prior to the implementation of the outpatient cost-sharing policy, as outpatient care at formal hospitals was not reimbursed, households confronted with minor illnesses or common diseases in children may have been more inclined to purchase medication directly from nearby pharmacies—a practice known as “self-medication.” The outpatient cost-sharing policy is theoretically associated with a lower relative price of formal medical services. Consequently, it is possible that parents might be less inclined to purchase medication from pharmacies and more likely to take their children to hospital outpatient departments. Thus, its association with better access to formal care may be through altering care-seeking behavior, rather than expanding the pool of the “paying population”, as sick children inevitably incur costs.

## 4. Discussion

This study evaluates the association between outpatient cost-sharing and rural child health and economic outcomes. Regarding the analysis of multiple outcomes, we view health, expenditure, and utilization as distinct dimensions based on a specific theoretical framework. Therefore, we consider these results to exhibit internal consistency and present unadjusted estimates. Key findings include:

First, the policy is significantly associated with better health scores and an increase in probability of outpatient visits. This may suggest that rural households are price-sensitive. The significant increase in reimbursement probability could indicate that the policy’s value lies in covering high-frequency, common outpatient expenses. We also find that the positive associations between the policy and child health outcomes appear to be connected to better mother’s health and greater school-related food and accommodation expenses.

Second, a key economic finding of this study is the possible rigid nature of demand for pediatric inpatient care. Unlike studies focusing on the elderly or patients with chronic diseases [34,35], we found the association of the policy variable with the probability of outpatient service utilization is significant, while the association with the probability of inpatient service utilization is statistically insignificant, which possibly means no substitution for children. This highlights the unique economics of pediatric care. As mentioned earlier, the reason for this difference may lie in the acute nature of childhood illness and the agency model of pediatric medical decision-making.

From an international perspective, this finding also contrasts with conclusions based on developed countries. Those studies might view greater outpatient use without decreased inpatient use as inefficient, because the goal of cost containment has not been achieved. However, in the context of rural China’s “under-utilization”, this represents a positive outcome. It suggests that the policy may be associated with the fulfillment of necessary demand and more comprehensive early intervention, which means better service balance.

Third, these findings have profound implications for rural sector. In the rural context, primary healthcare institutions are the main providers of outpatient services. This policy may be associated with a tendency for rural pediatric patients to utilize local clinics due to reimbursement incentives, which is the key for the issue of imbalanced resource allocation under the dual structure. This could indicate that for rural China, this policy is not only a financial tool, but also a means to potentially optimize the allocation of scarce pediatric resources.

Furthermore, while cross-sectional data limit our ability to trace long-term outcomes directly, our empirical results could imply potential long-term benefits. For instance, the policy is associated with higher household investment in child school-related food and accommodation expenses. According to the theory of early human capital formation, school-age health inputs are foundational for future cognitive development [36].

Limitations: We acknowledge that reliance on cross-sectional data limits the ability to draw definitive causal conclusions. Therefore, the relationships identified should be interpreted as associations rather than strictly causal effects. Future research should utilize panel data or quasi-experimental designs to achieve causal inference and explore long-term human capital effects.

## 5. Conclusions

This study provides new evidence that the outpatient cost-sharing policy is associated with better health for rural Chinese children. It also explores the potential factors by discussing mother health, school-related food and accommodation expenses, and medical-seeking behavior. Uniquely, we find pediatric inpatient demand is rigid, challenging the “outpatient-substituting-inpatient” logic derived from adult studies.

Our findings are consistent with some other international evidence. For instance, Kang et al. (2022) found that in Japan, lower co-payments improved children’s health status and increased outpatient utilization without reducing inpatient care [37], suggesting that substitution is not the primary driver. Evidence from diverse contexts, including China, Pakistan, and the U.S., indicates that health insurance promotes child health via multifaceted pathways, including enhanced maternal health and increased spending on children’s food [38,39,40].

Based on the observed associations, we suggest the following policy implications for consideration to further optimize China’s rural child medical security system:

Consider Broader Implementation: The positive associations observed in this study suggest that the policy aligns well with children’s “outpatient-heavy” healthcare needs, which may inform policymakers to expand the policy to include more rural children.

Refine Evaluation Metrics: Our findings suggest that traditional metrics focusing solely on cost containment or inpatient substitution may not fully capture the policy’s value for children. Future evaluations might benefit from prioritizing metrics such as health outcomes and accessibility.

## Figures and Tables

**Table 1:** Descriptive Statistics of Variables.

Group	Variables	Full Sample				Treated Sample	Control Sample
		Mean	Std	Min	Max	Mean	Mean
Dependent Variables	Child Health Score	4.485	0.628	1	5	4.546	4.433
	Out-of-pocket Med-Expenses	2.661	2.926	0	11.002	2.731	2.62
	Reimbursement Ratio	8.919	19.648	0	90.909	10.029	6.776
Control Variables	Age	7.968	3.667	0	14	7.829	8.084
	Gender	0.527	0.499	0	1	0.532	0.523
	Birth Weight	6.327	1.062	2	12	6.299	6.35
	Mother’s Employment	0.745	0.435	0	1	0.776	0.721
	HH Per Capita Income	1.148	1.155	0.002	28.029	1.389	0.948
	Hospital Beds	3.638	0.337	2.96	4.606	3.716	3.574
Channels Variables	Mother’s Health	4.255	0.785	1	5	4.389	4.156
	School-related Food and Accommodation Expenses	3.971	3.634	0	10.555	3.786	4.124
	Inpatient or Not	0.017	0.129	0	1	0.012	0.021
	Outpatient or Not	0.218	0.413	0	1	0.261	0.183

**Table 2:** Association of outpatient cost-sharing Policy with Rural Children’s Health.

	Child Health Score	
	Oprobit	PSM-Oprobit
	(1)	(2)
policy	0.246 (0.160)	0.307 ** (0.153)
samples	3122	3119

Note: The city-clustered standard errors are shown in bracket, with *, **, and *** indicating significant at the 10%, 5%, and 1% levels, respectively.

**Table 3:** Association of outpatient cost-sharing Policy with Rural Children’s Medical Service Expenditure and Reimbursement Ratio.

	Medical Service Expenditure		Medical Service Reimbursement Ratio	
	(1)	(2)	(3)	(4)
	The First Part	The Second Part	The First Part	The Second Part
policy	−0.050 (0.221)	−0.257 (0.186)	0.594 ** (0.300)	0.294 (4.982)
samples	2988	1430	1430	318

Note: The city-clustered standard errors are shown in bracket, with *, **, and *** indicating significant at the 10%, 5%, and 1% levels, respectively.

**Table 4:** Association of Outpatient Cost-sharing Policy with Rural Children’s Medical-Seeking Behavior, Mother’s Health and School-related Food and Accommodation Expenditure.

	Mother’s Health		Outpatient or Not		Inpatient or Not		School-Related Food and Accommodation Expenditure	
	(1)	(2)	(3)	(4)	(5)	(6)	(7)	(8)
	Oprobit	PSM-Oprobit	Logit	PSM-Logit	Logit	PSM-Logit	The First Part	The Second Part
policy	0.248 ** (0.104)	0.202 * (0.106)	0.318 (0.210)	0.347 * (0.205)	−0.210 (0.474)	−0.009 (0.489)	−0.155 (0.136)	0.232 ** (0.106)
samples	2146	2141	3106	3103	3106	3103	2612	1454

Note: The city-clustered standard errors are shown in bracket, with *, **, and *** indicating significant at the 10%, 5%, and 1% levels, respectively. All PSM models have passed balance test.

## Data Availability

The original data is public secondary data, and can be accessed through the Chinese Household Income Project (CHIP) at the following links: https://bs.bnu.edu.cn/zgjmsrfpdcsjk/sjsq/index.html (accessed on 21 August 2025) with the permission of the Business School at Beijing Normal University.

## Appendix A

**Table A1:** Cities and Implementation Year that Had Implemented the URRBMI Outpatient Cost-sharing Policy by the 1 January 2018 in China.

City Names	Year	City Names	Year	City Names	Year	City Names	Year
Yingkou	2008	Yunfu	2012	Zibo	2016	Wuhan	2017
Datong	2009	Meishan	2012	Weihai	2016	Hengyang	2017
Wuxi	2010	Zhenjiang	2013	Chenzhou	2016	Yongzhou	2017
Hefei	2010	Kunming	2013	Nanchong	2016	Shantou	2017
Ezhou	2010	Suqian	2014	Ya’an	2016	Lianyungang	2018
Enshi	2010	Binzhou	2014	Taiyuan	2017	Yancheng	2018
Guangzhou	2010	Meizhou	2014	Bayannur	2017	Dezhou	2018
Maoming	2010	Suining	2014	Xuzhou	2017	Yichang	2018
Qingyuan	2010	Bazhong	2014	Huai’an	2017	Xiangyang	2018
Leshan	2010	Nantong	2015	Yangzhou	2017	Neijiang	2018
Zhaoqing	2011	Jinan	2015	Taizhou	2017	Baiyin	2018
Chengdu	2011	Qingdao	2015	Zhengzhou	2017		
Dongying	2012	Weifang	2015	Xinxiang	2017		
Xianning	2012	Yiyang	2015	Xuchang	2017		
Jieyang	2012	Suzhou	2016	Xinyang	2017		

**Table A2:** Comparison of Characteristics between Control Group and Treatment Group.

Variables	Mean (Control)	Mean (Treatment)	Diff
Age	8.084	7.829	0.255
Gender	0.523	0.532	−0.009
Birth Weight	6.35	6.299	0.051
Mother’s Employment	0.721	0.776	−0.055 ***
HH Per Capita Income	0.948	1.389	−0.442 ***
Hospital Beds	3.574	3.716	−0.142 ***

Note: *, **, and *** indicate significant at the 10%, 5%, and 1% levels, respectively.

**Table A3:** First-Stage Logit Model Estimation of Propensity Scores.

Variables	Coeff.
Age	−0.027 ***
Gender	0.033
Birth Weight	−0.099 ***
Mother’s Employment	0.191 **
HH Per Capita Income	0.503 ***
Hospital Beds	1.078 ***
Pseudo R2	0.064

Note: *, **, and *** indicate significant at the 10%, 5%, and 1% levels, respectively.

**Table A4:** Robustness Check Results.

	Sample Sensitivity Analysis			PSM-OLS	Tobit	
	(1)	(2)	(3)	(4)	(5)	(6)
	Health Score	Medical Service Expenditure	Reimbursement Ratio	Health Score	Medical Service Expenditure	Reimbursement Ratio
policy	0.296 * (0.153)	−0.074 (0.219)/ −0.255 (0.186)	0.594 ** (0.299)/ 0.291 (4.989)	0.152 ** (0.076)	−0.264 (0.582)	0.158 * (0.083)
samples	2810	2824/ 1303	1303/ 314	3122	2988	1430

Note: The two rows of coefficients in columns 2 and 3 of the table represent the coefficients of the first and second parts of the Two-Part Model, respectively. The city-clustered standard errors are shown in bracket, with *, **, and *** indicating significant at the 10%, 5%, and 1% levels, respectively.
